# Perioperative/Periprocedural Antithrombotic Management in Oral Health Procedures. A Prospective Observational Study

**DOI:** 10.3390/dj13050196

**Published:** 2025-04-29

**Authors:** María González-Zamora, Nagore Ambrosio, Raquel González, Paula Anguita, Ana Molina, David Herrera, Mariano Sanz, Francisco Marín, María Anguita-Gámez, Raquel Ferrandis, David Vivas, Manuel Anguita, Elena Figuero

**Affiliations:** 1 PhD Student, Department of Dental Clinical Specialties, Complutense University of Madrid (UCM), 28040 Madrid, Spain; mgonza51@ucm.es; 2 ETEP (Etiology and Therapy of Periodontal and Peri-implant Diseases) Research Group, Complutense University of Madrid (UCM), 28040 Madrid, Spain; ar.molina@ucm.es (A.M.); marsan@ucm.es (M.S.); elfiguer@ucm.es (E.F.); 3 Postgraduate Specialization Program in Periodontology, Department of Dental Clinical Specialties, Complutense University of Madrid (UCM), 28040 Madrid, Spain; raqgon09@ucm.es; 4 Faculty of Health Sciences, HM Hospitals, Camilo José Cela University, 28692 Madrid, Spain; paula.anguita@ucjc.edu; 5 Cardiology Unit, Virgen de la Arrixaca University Clinical Hospital, 30120 Murcia, Spain; fr.marino@um.es; 6 CIBER Cardiovascular, Instituto San Carlos III, 28029 Madrid, Spain; manuelanguita@secardiologia.es; 7 Cardiovascular Institute, San Carlos Clinical Hospital, 28040 Madrid, Spain; maanguit@ucm.es (M.A.-G.); dvivas@secardiologia.es (D.V.); 8 Anesthesiology and Critical Care Department, Hospital Universitari i Politècnic La Fe, Universitat de València, 46010 Valencia, Spain; raquel.ferrandis@uv.es; 9 Department of Medicine, Faculty of Medicine, Complutense University of Madrid (UCM), 28040 Madrid, Spain; 10 UGC Cardiology, Reina Sofía University Hospital, IMIBIC, University of Córdoba, 14004 Córdoba, Spain

**Keywords:** antithrombotic, anticoagulants, antiplatelets, oral health procedures, peri/postprocedural complications

## Abstract

**Background/Objectives:** This paper evaluates the incidence of thrombotic and/or hemorrhagic adverse events within 30 days after oral health procedures (OHPs) in patients taking antithrombotic agents. Secondary objectives were to determine proper antithrombotic management and its association with adverse events. **Methods:** As part of a multicenter multispecialty prospective observational study (ReQXAA), individuals with antithrombotic therapy and receiving at least one OHP were selected. Before OHP, participants were referred to their medical doctors to indicate the antithrombotic therapy management. Adverse events were evaluated thirty days after OHP by phone call. Proportions and odds ratios (ORs) were generated applying Fisher’s exact test, chi-square tests and multiple regression models. **Results:** A total of 138 patients underwent 144 OHPs. Fifteen adverse events (10.5%) were registered, among which the most frequent was slight bleeding (n = 13), which was followed by bleeding that required suspension of the antithrombotic agent (n = 1) and a myocardial infarction (n = 1). Antithrombotic management was appropriate in 122 (84.7%) cases. In 15.3% of the cases it was inappropriate, the main reason being the unnecessary interruption of the antithrombotic medication (n = 11; 50%). Inadequate management was associated with a higher incidence of adverse events (OR = 4.7; 95% confidence interval [1.3, 16.3]; *p* = 0.016) after adjusting for confounding factors. **Conclusions:** The incidence of adverse events 30 days after OHPs was low (10.5%). An inappropriate perioperative/periprocedural antithrombotic management occurred in 15.3% of the cases and was associated with a higher incidence of adverse events (OR = 4.7).

## 1. Introduction

The increasing age of the global population, coupled with a higher incidence of cardiovascular diseases and the advances in medical care of acute cardiovascular events, has led to a population of chronic cardiovascular patients receiving antithrombotic therapy [1]. These antithrombotic medications, including antiplatelet agents and anticoagulants, are critical in the prevention and mitigation of thromboembolic complications, such as myocardial infarction, stroke, and deep vein thrombosis. In Spain, over 800,000 patients, primarily suffering from atrial fibrillation, are taking anticoagulants [2]. Similarly, the use of antiplatelet agents in the secondary prevention of cardiovascular events, mainly after post-percutaneous coronary interventions and stent implantation, has increased [3]. In addition, there are drugs other than antithrombotic, such as antidepressants and complementary medicines that also impair hemostasis [4]. Dental interventions in these patients can be challenging, as the risk of bleeding from the continuation of antithrombotic therapy needs to be weighed against the thromboembolic risk associated with drug interruption or de-escalation [5]. Consequently, oral health professionals encounter a growing number of patients using a variety of antithrombotic regimens, which demands from these professionals a clear understanding of their pharmacological implications as well as their association with possible complications during and after the oral health procedures (OHPs). Furthermore, oral health professionals should be trained on the perioperative management strategies associated to the use of these medications [6].

Oral health procedures affected by the use of antithrombotic medications may range from simple professional mechanical plaque removal (PMPR) to complex surgical interventions, which demands standardized management approaches to mitigate bleeding complications without compromising the cardiovascular risk control.

**Figure 1:**
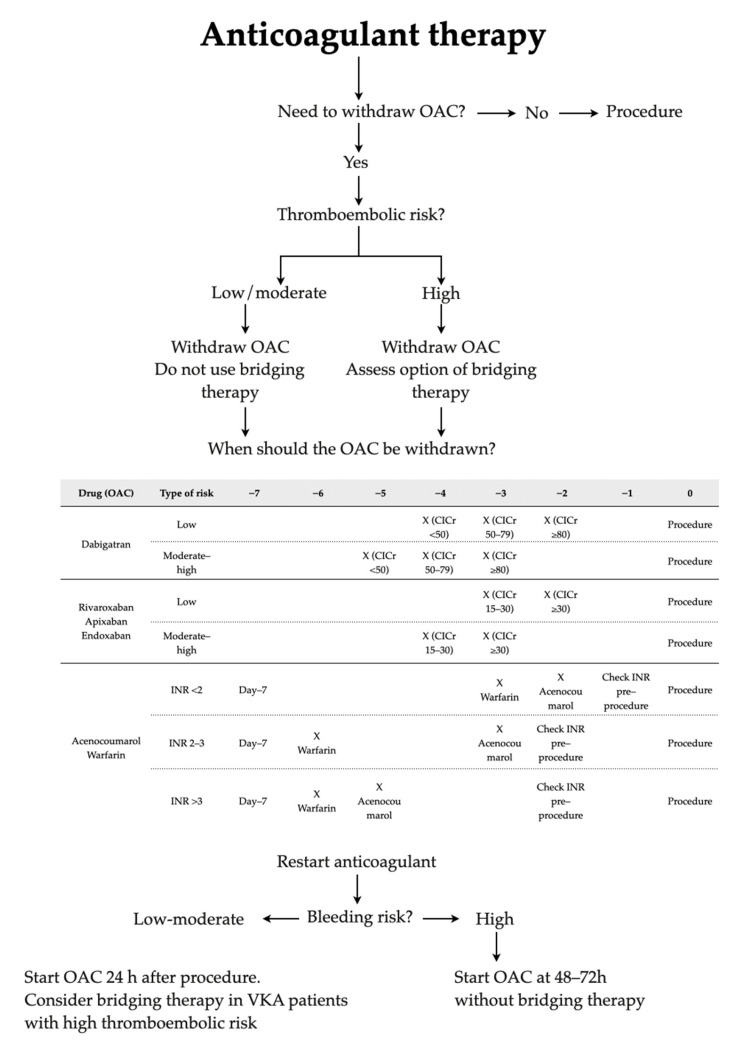
Management of anticoagulant therapy. Adapted from Vivas et al. 2018 [3].

**Figure 2:**
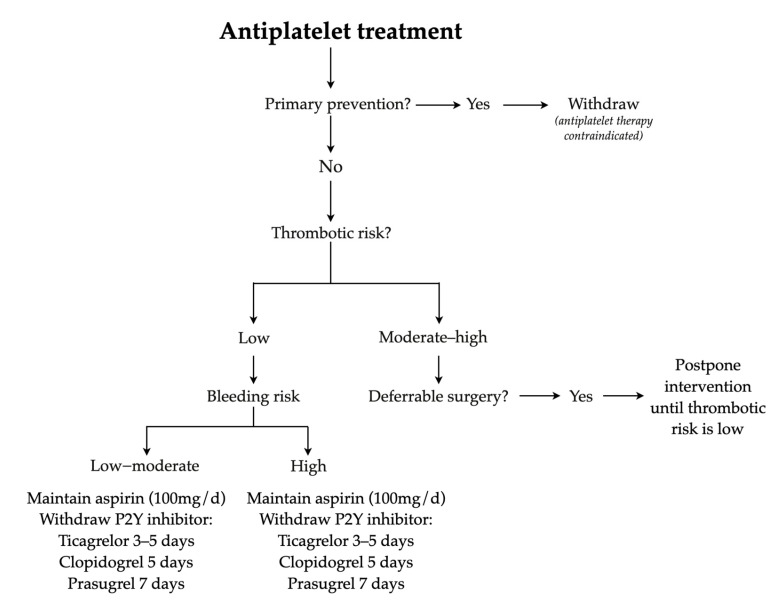
Management of antiplatelet treatment. Adapted from Vivas et al. 2018 [3].

Despite the existence of standard recommendations in the literature, their implementation for oral health procedures in dental clinics remains limited [7]. The current recommendations for the management of antithrombotic medications are based on the assessment of the patient thrombotic risk and the hemorrhagic risk of the intervention [3]. A consensus document endorsed by numerous Spanish scientific societies in 2018 addressed the perioperative/periprocedural management of antithrombotic agents [3]. This consensus advised maintaining the use of acetylsalicylic acid (ASA) unless contraindicated for the antiplatelet management and suggested that decisions on other medications should depend on thrombotic risk. Additionally, it is recommended not to interrupt the anticoagulation medication for low-risk procedures where the possible bleeding was clinically manageable (Figure 1 and Figure 2). After the publication of the consensus, an observational study was conducted to assess the determine the adherence to these recommendations. Consequently, in 2023, the Spanish registry REQXAA (“Prospective observational registry of perioperative and periprocedural management of antithrombotic therapy in real world”) revealed that only 57.3% of the cases were adequately managed, and the inappropriate management is an independent risk factor for thrombotic and hemorrhagic events [8].

The present study is a secondary analysis of this ReQXXA study [8] focusing solely on individuals undergoing OHP. The primary objective was to analyze the incidence of thrombotic and/or hemorrhagic adverse events during the initial 30 days post-OHP. The secondary objectives were to evaluate adherence to the consensus document on the perioperative/periprocedural management of antithrombotic agents [3] undergoing OHP and to determine its association with thrombotic and/or hemorrhagic adverse events (30 days post-OHP).

## 2. Materials and Methods

### 2.1. Study Design

This study was part of a multicenter multispecialty prospective observational study with patients in antithrombotic treatment requiring different interventions. The main objective was to analyze the incidence thrombotic and/or hemorrhagic adverse events (30 days post-OHP) [8]. In this subsample analysis, individuals taking antithrombotic agents and receiving at least one OHP with risk of bleeding (restorative interventions, subgingival instrumentation, supportive periodontal care visit, tooth extraction, periodontal or peri-implant surgery, implant placement surgery, sinus lift surgery, second phase implant surgery, apical surgery, pre-prosthetic surgery and bone regeneration surgery) were selected mainly from the Clinic of Postgraduate Specialization in Periodontology at the Complutense University of Madrid, as well as from six additional locations, including hospitals and primary care health centers. The protocol was in accordance with the Helsinki Declaration (2008) and has been previously approved by the Ethics Committee of Hospital Clínico San Carlos (19/440-E, approved on 28 October 2019). All patients signed an informed consent form to participate after being informed of the characteristics of this clinical study and given the possibility to withdraw from the study.

### 2.2. Study Population

Patients were consecutively selected among those under chronic treatment with antithrombotic agents and scheduled for an OHP. They were selected based on the following inclusion and exclusion criteria.

#### 2.2.1. Inclusion Criteria

Patients were included if they were (1) at least 18 years-old, (2) in treatment with at least one antithrombotic agent, either antiplatelet and/or anticoagulant, and (3) receiving OHPs with risk of bleeding.

#### 2.2.2. Exclusion Criteria

Patients were excluded if it was not possible to contact them for a follow-up of 30 days after the OHPs.

### 2.3. Sample Size

The original study aimed to detect 50% lesser incidence in adverse events when comparing patients with or without appropriate management of the antithrombotic therapy. To achieve this objective, 1000 patients were initially planned, although 1266 individuals were finally included [8]. In the present secondary analysis focusing on OHPs, participants receiving only OHPs were selected from the original sample.

### 2.4. Study Visits

Each patient’s eligibility was evaluated at the dental clinic during an initial in-person screening visit and according to the aforementioned criteria. If included, patients were referred to their medical doctor (general practitioner or specialist) with a report to establish what the management of antithrombotic treatment should be in relation to the proposed OHP. At a subsequent visit, the patients underwent the planned OHP, which was followed by a phone call 30 days later to gather information on the occurrence of any adverse events.

### 2.5. Study Outcomes

The primary outcome was the incidence of any thrombotic and/or hemorrhagic adverse event within the 30 days post-OHP.

Furthermore, the following secondary variables were self-reported registered:

- *Demographic data*: gender and age.
- *Systemic status*: smoking habit, height, weight and pre-existing systemic pathologies. Body mass index [BMI] was later calculated by the researchers.
- *Medication intake*: reason for the antithrombotic therapy, type of antithrombotic therapy (categorized as antiplatelet, anticoagulant and double medication), and adherence of the prescribed antithrombotic regime to the REQXXA consensus document [3], as established using the QXAApp web application (https://qxaapp.secardiologia.es/farmacos; accessed on 5 April 2025) and rated as appropriate or inappropriate. An inappropriate management was considered as any deviation from the recommendation (e.g., unnecessary interruption, lack of a necessary interruption, incorrect timing or unnecessarily prescription of bridging therapy) [3].
- *Patient thrombotic risk*: categorized as high, moderate and low based on the annual risk of arterial or venous thromboembolism according to duration of treatment and reason for antiplatelet therapy (acute coronary syndrome, stable coronary disease, cerebrovascular disease and peripheral artery disease) [9].
- *Bleeding risk:* stratified into 3 levels according to the characteristics of the procedure the patient is to undergo. Procedures with a low bleeding risk are those in which adequate hemostasis can be achieved and in which bleeding would not jeopardize the patient’s life, affect the outcome of surgery, or require transfusion. Procedures with a moderate bleeding risk, in turn, are those in which it may be difficult to secure hemostasis or in which bleeding would increase the likelihood of the need for a transfusion or a repeat operation. Finally, procedures with a high bleeding risk are those in which perioperative bleeding could place the patient’s life at risk or compromise the outcome of surgery [3].
- *Oral health procedure*: type of dental intervention received (categorized as restorative, non-surgical periodontal interventions [PMPRs, subgingival instrumentation, supportive periodontal care], tooth extractions and surgical interventions [periodontal or peri-implant surgery, implant placement surgery with or without simultaneous bone regenerative procedure], sinus lift surgery, second phase implant surgery, apical surgery, pre-prothesis surgery and bone regeneration surgery) and hemorrhagic risk of the procedure (classified as low, moderate or high based on the likelihood of achieving hemostasis and its potential impact on patient outcomes) [3].
- *Adverse events:* categorized using the criteria from the Bleeding Academic Research Consortium (BARC) [10], including myocardial infarction, stent thrombosis, ischemic stroke, venous thromboembolic disease as well as various bleeding scenarios and hemorrhagic complications.

### 2.6. Statistical Analysis

Patient and intervention-level analyses were performed. Demographic and systemic status data (including gender, age, smoking habit, BMI, and pre-existing systemic pathologies), thrombotic risk, type of antithrombotic therapy and reason for antithrombotic therapy were analyzed at the patient level. All other variables were assessed by intervention-level analysis.

For quantitative outcomes, normality was assessed using the Kolmogorov–Smirnov test and box-plots, and results were presented as mean and standard deviations (SDs). Categorical outcomes were described using proportions and contingency tables with application of Fisher’s exact or chi-square test.

A multiple binary logistic regression model was employed to assess the impact of the adherence to the consensus report [3] on the incidence of adverse events, adjusting for confounding factors determined by theoretical plausibility and the statistical significance attained in the univariate analysis (*p* < 0.20). Confounders were added to the model (≥75 years old, gender, and type of dental intervention) one by one, and only those causing clinically significant change in the odds ratio (OR) (10%) were included in the adjusted model. Results were expressed using OR and its 95% confidence interval (CI).

The level of statistical significance was set at *p* < 0.05. All analyses were carried out using IBM SPSS Statistics 28.0.0.0 (IBM Corporation, Armonk, NY, USA).

## 3. Results

### 3.1. Sample Description

The characteristics of the overall study sample have already been published [8]. The present study reports a subsample of 138 patients from seven different centers, which is depicted in Table S1. The mean age was 68.1 years (SD = 10.2), being 53.6% of males (Table 1). Thrombotic risk was low in 119 (86.2%) of the patients, moderate in 16 (11.6%) and high in 3 (2.2%). The hemorrhagic risk was low in 137 (99.3%) patients and moderate in 1 (0.7%) patient; none had high risk.

Overall, 79 patients (57.2%) were under treatment with antiplatelet agents, 51 (37.0%) with anticoagulants and 8 (5.8%) with a combined therapy (Table 2). A distinction was made between concomitant conditions and the primary condition for which the patient is receiving medication. The most common reason for receiving antithrombotic therapy was ischemic heart disease (n = 47; 36.2%), which was followed by atrial fibrillation (n = 36; 27.7%) and primary prevention of cardiovascular disease (n = 22; 16.9%) (Table S2).

One hundred and forty-four OHPs were performed in 138 patients (Table 3), with most of them receiving a single OHP, except for six patients who received two different OHPs and one patient that received three different OHPs. The information on each individual OHP and the type of antithrombotic therapy are described in Table 4 and Table S3.

### 3.2. Antithrombotic Therapy Management

Following the consensus report guidelines [3], antithrombotic therapy management was considered appropriate in 122 (84.7%) OHPs and inappropriate in 22 (15.3%) OHPs. The reasons for inappropriate management were mainly related to an unnecessary interruption of the antithrombotic medication (n = 11; 50%), the lack of a necessary interruption (n = 8; 36.4%) of the antithrombotic medication, an unnecessarily prescription of bridging therapy (n = 2; 9.1%) and an incorrect timing (n = 1; 4.5%).

The inappropriate antithrombotic management was not statistically associated with the received OHP (either categorized (*p* = 0.058; Table 5) or individually (*p* = 0.146; Table S4). However, the suspension of the antithrombotic treatment was significantly associated with the category of OHP (*p* = 0.002) and with individual OHP (*p* = 0.01), and tooth extraction was the most common OHP where medication was suspended (n = 13; 38.2%), which was followed by implant surgery (n = 8; 29.6%).

### 3.3. Incidence of Adverse Events

Overall, 15 adverse events (10.5%) occurred in 15 patients receiving one OHP, among which the most common the occurrence of slight post operatory bleeding (n = 13;), which was followed by post-operatory bleeding requiring suspension of the antithrombotic therapy (n = 1) and post-operatory incidence of a myocardial infarction (n = 1). This myocardial infarction occurred in a patient on antiplatelet therapy (acetylsalicylic acid), which was inappropriately discontinued 5 days before the procedure.

Events were evenly distributed among patients on different antithrombotic agents. Individual OHPs were associated with event occurrence (*p* = 0.048), particularly minor bleeding in dental extractions, subgingival instrumentation, periodontal surgery, and sinus lift floor elevation (Table S5).

The association of adverse events with age (≥75 years old), gender, cardiovascular risk factors (smoker, hypertension and diabetes mellitus), OHP, type of antithrombotic therapy, inappropriate antithrombotic management and hemorrhagic/thrombotic risk of patients is described in Table 6.

A statistically significant association was observed between the incidence of adverse events and inappropriate antithrombotic management (*p* = 0.002). This association was further explored by a regression logistic model demonstrating an OR = 6.7 (95% CI [2.1; 21.0], *p* = 0.01) (Table 7). The addition of confounders to the model as potential sources of variability (age ≥75 years old, gender and type of OHP) resulted in an OR of 4.7 (95% CI [1.33; 16.33], *p* = 0.016).

## 4. Discussion

The results from the present study are based on 138 patients taking antithrombotic medication and undergoing at least one OHP. In this sample, the incidence of adverse (thrombotic and/or hemorrhagic) events within 30 days post-OHP was 10.5% (n = 15 patients). When the appropriateness of the antithrombotic management using standard guidelines was analyzed, in 15.3% of the patients, this management was inappropriate, which was associated with a higher incidence of adverse events (OR = 4.7).

This reported incidence of adverse events in patients taking antithrombotic medication and undergoing an OHP is in accordance with the data from the original study reporting an incidence of 7.6% (96 patients) considering a composite clinical event within the first 30 days after the intervention [8]. However, the type of event differed depending on the associated clinical intervention: a slight bleeding was the most commonly reported adverse event after OHP (n = 13; 9.0%), while 27 cases of death were reported in the overall original study, which included other different type of medical surgical procedures. It is worth noting that patients who underwent surgical procedures such as tooth extractions or surgeries received sutures to promote hemostasis. After experiencing bleeding, the most common hemostatic measure was compression with gauze and the topical application of tranexamic acid 5% (Amchafibrin^®^ 500 mg tablets 5 mL; Rottapharm Madaus S.L., Barcelona, Spain).

In the present study, only two major events were reported (one patient experienced bleeding that required suspension of antithrombotic therapy, and another patient suffered a myocardial infarction). Regarding this latter event, it occurred after a restorative procedure (low bleeding risk) where the antiplatelet medication (acetylsalicylic acid) was inappropriately interrupted five days prior to the OHP in a patient with a moderate thrombotic risk. This finding should be highlighted, considering the large number of patients on antithrombotic treatment who require an OHP at some point in their lives, in which the occurrence of a severe medical complication is not commensurate with the type of OHP. A similar event occurred in a clinical trial to evaluate the effect of tranexamic acid, where one patient in the placebo group had a transient ischemic attack while interrupting the DOAC therapy in preparation for the dental extraction [11].

The present study also reports a low rate (15.3%) of non-adherence to the consensus document on perioperative/periprocedural management of antithrombotic agents [3] compared with the 42.7% rate of inappropriate antithrombotic management reported in the overall study [8]. This consensus document [3] recommends the periprocedural maintenance of acetylsalicylic acid in almost all interventions, and the consideration to also maintaining a dual antiplatelet treatment in surgeries with low hemorrhagic risk. In line with this consensus document, the current AHA/ACC/SCAI/ACS/ADA/ESC/ACCP guidelines on the perioperative management of antithrombotic therapy do not recommend the discontinuation of antiplatelet therapy for low bleeding risk procedures [12,13,14]. Vivas et al. [3] recommended the avoidance of discontinuing the anticoagulant therapy for low-risk interventions, since the bleeding risk is considered minimal and acceptable by the operator [15]. Additionally, the consensus document advises minimizing the suspension of anticoagulant treatment based on the pharmacokinetics of the antithrombotic, reserving bridging therapy for high thromboembolic risk patients. Bridging therapy refers to the temporary suspension of the anticoagulant, which is replaced by short-acting parenteral anticoagulants, such as unfractionated heparins or low molecular weight heparins. Depending on the risk of thrombosis—high (>10%), moderate (5–10%), and low (≤5%)—the eventual need for bridge therapy was determined [9]. Although antithrombotic agents cause an increase in bleeding, there is general consensus that treatment regimens should not be altered before routine dental procedures when the risk of bleeding is moderate to low [16]. An expert group report recommends that dental practitioners should seek appropriate advice and consult with the responsible medical professional when the international normalized ratio (INR) is greater or equal to 3.5 [17]. In the present study, INR levels were not known prior to the intervention in all patients. Previous studies did not report increased bleeding rates with continued warfarin therapy during dental extractions [18,19]. The findings from the present study agree with these data since slight bleeding only occurred in seven patients undertaking anticoagulants (13.0%). Also, they are in agreement with a retrospective study evaluating the management of direct oral anticoagulants in older patients undergoing dental extraction that reported postoperative bleeding being more frequent in elderly patients [20]. It is worth noting that hemostatic agents are effective in reducing the time to achieve hemostasis in patients undergoing tooth extractions [21]. The topical hemostatic agents play an important role as useful adjuncts to prevent further bleeding [22].

The strength of this observational study is the identification of a significant association between the incidence of adverse effects and the inappropriate management of antithrombotic medication in OHPs. However, the study has also clear limitations due to the observational nature of this study design, and hence, the study only assessed adverse events within 30 days post-OHP. A longer follow-up period could provide additional insights into the long-term outcomes of antithrombotic management. Another issue was the lack of randomization of the patients to specific perioperative antithrombotic treatment regimens. Furthermore, adverse events were self-reported, and the sample size could be considered small because it is a subsample analysis. Nearly 30% of the patients underwent subgingival instrumentation, which can be considered a low-risk procedure for prolonged bleeding compared to other surgical interventions. Consequently, caution should be taken in the interpretation of the results concerning the incidence of adverse events and the impact of an inappropriate management of the antithrombotic medication.

While this study provided short- to medium-term information, further longitudinal research is required to understand the long-term impact of the perioperative management of antithrombotic agents in a dental setting. These studies could be used to develop predictive models that would generate individualized treatment strategies.

## 5. Conclusions

The present study reports an incidence of 10.5% adverse (thrombotic and/or hemorrhagic) events during the initial 30 days after an OHP in patients on antithrombotic medication. During these OHP procedures, an inappropriate antithrombotic management occurred in 15.3% of the cases, which was associated with a higher incidence of adverse events (OR = 4.7).

## Figures and Tables

**Table 1:** Patient demographics and systemic health information.

**Age** (years) *(mean [SD])*	68.1 (10.2)
**Sex**	
Male *(n [%])*	74 (53.6)
**Smoking habit**	
Smokers *(n [%])*	32 (23.2)
**BMI** *(mean [SD])*	27.36 (3.8)
Pre-existing systemic pathologies * *(n [%])*	
Hypertension	92 (66.7)
Diabetes mellitus	42 (30.4)
Hypercholesterolemia	76 (55.1)
Stroke/transient ischemic attack	38 (27.5)
Ischemic heart disease	58 (42.0)
Pulmonary embolism	1 (0.7)
Deep venous thrombosis	12 (8.7)
Peripheral arterial disease	23 (16.7)
Heart failure	26 (18.8)
Chronic kidney disease	4 (2.9)
Cancer	7 (5.1)
COPD	5 (3.6)
Liver disease	1 (0.7)
Alcohol abuse, enolism	1 (0.7)
Anemia	2 (1.4)
Thrombophilia	0 (0)
Bleeding episodes	8 (5.8)
**Thrombotic risk** *(n [%])*	
Low	119 (86.2)
Moderate	16 (11.6)
High	3 (2.2)
**Hemorrhagic risk** *(n [%])*	
Low	137 (99.3)
Moderate	1 (0.7)
High	0 (0)

BMI: body mass index; COPD: chronic obstructive pulmonary disease; SD: standard deviation. Pre-existing systemic pathologies refer to concomitant pathologies *.

**Table 2:** Distribution and frequency of antithrombotic therapy.

	n (%)
**Antiplatelet**	79 (57.2)
Acetylsalicylic acid (ASA)	70 (50.7)
Clopidogrel	9 (6.5)
**Anticoagulant**	51 (37.0)
Acenocoumarin	22 (15.9)
Dabigatran	10 (7.2)
Rivaroxaban	2 (1.4)
Apixaban	9 (6.5)
Edoxaban	8 (5.8)
**Combination**	8 (5.8)
ASA and Edoxaban	1 (0.7)
ASA and Acenocoumarin	1 (0.7)
ASA and Ticagrelor	3 (2.2)
ASA and Clopidogrel	3 (2.2)

**Table 3:** Distribution and frequency of oral health procedures.

	n (%)
**Restorative interventions**	4 (2.8)
**Non-surgical periodontal interventions**	55 (38.2)
Subgingival instrumentation	43 (29.9)
Supportive periodontal care visit	12 (8.3)
**Tooth extraction**	34 (23.6)
**Surgical interventions**	51 (35.4)
Periodontal or peri-implant surgery	15 (10.4)
Implant placement surgery	27 (18.7)
Sinus lift surgery	4 (2.8)
Second phase implant surgery	1 (0.7)
Apical surgery	1 (0.7)
Pre-prosthetic surgery	1 (0.7)
Bone regeneration surgery	2 (1.4)

**Table 4:** Frequencies and prevalence of oral health procedures and the type of antithrombotic therapy.

(a) Main antithrombotic therapy					
	Restorative	Non-Surgical Periodontal Interventions	Tooth Extraction	Surgical Interventions	*p Value*
**Antiplatelet (n [%])**	3 (75.0)	38 (69.1)	17 (50.0)	24 (47.1)	0.037
**Anticoagulant (n [%])**	1 (25.0)	17 (30.9)	15 (44.1)	21 (41.2)	
**Combination (n [%])**	0 (0)	0 (0)	2 (5.9)	6 (11.8)	
**(b) Individual antithrombotic therapy**					
	**Restorative**	**Non-Surgical Periodontal Interventions**	**Tooth** **Extraction**	**Surgical** **Interventions**	***p Value***
**Antiplatelet (n [%])**					0.003
Acetylsalicylic acid (ASA)	2 (50.0)	34 (61.8)	15 (44.1)	21(41.2)	
Clopidogrel	1 (25.0)	4 (7.3)	2 (5.9)	3 (5.9)	
**Anticoagulant (n [%])**					
Acenocoumarin	0 (0)	9 (16.4)	5 (14.7)	9 (17.6)	
Dabigatran	1 (25.0)	1 (1.8)	5 (14.7)	5 (9.8)	
Rivaroxaban	0 (0)	1 (1.8)	1 (2.9)	0 (0)	
Apixaban	0 (0)	4 (7.3)	3 (8.8)	2 (3.9)	
Edoxaban	0 (0)	2 (3.6)	1 (2.9)	5 (9.8)	
**Combination (n [%])**					
ASA and Edoxaban	0 (0)	0 (0)	0 (0)	1 (2.0)	
ASA and Acenocoumarin	0 (0)	0 (0)	0 (0)	1 (2.0)	
ASA and Ticagrelor	0 (0)	0 (0)	2 (5.9)	1 (2.0)	
ASA and Clopidogrel	0 (0)	0 (0)	0 (0)	3 (5.9)	

**Table 5:** Antithrombotic therapy management in relation to the type of oral health procedure category.

	Restorative	Non-Surgical Periodontal Interventions	Tooth Extraction	Surgical Interventions	*p Value*
**Inappropriate antithrombotic management (n [%])**	2 (50)	4 (7.3)	8 (23.5)	8 (15.7)	0.058
*Unnecessary interruption (n [%])*	1 (50)	3 (75)	5 (62.5)	2 (25)	0.727
*Lack of a necessary interruption (n [%])*	1 (50)	1(25)	2 (25)	4 (0)	
*Incorrect timing (n [%])*	0 (0)	0 (0)	0 (0)	1 (12.5)	
*Unnecessarily prescription of bridging therapy (n [%])*	0 (0)	0 (0)	1 (12.5)	1 (12.5)	
**Suspension (n [%])**	2 (50)	4 (7.3)	13 (38.2)	13 (25.5)	0.002

**Table 6:** Association of adverse events with different outcome variables.

	Event (n [%])	*p Value*
**Antithrombotic management**		
Appropriate	8 (6.6)	0.002
Inappropriate	7 (31.8)	
**Age (≥75years old)**	6 (18.2)	0.195
**Gender**		0.108
Male	5 (6.8)	
Female	10 (15.6)	
**Cardiovascular risk factors**		
Smoker	4 (12.1)	0.930
Hypertension	11 (11.7)	0.577
Diabetes mellitus	6 (14.0)	0.381
**Oral health procedures**		0.120
Restorative	2 (50)	
Non-surgical periodontal	3 (5.5)	
Extraction	4 (11.8)	
Surgical	6 (11.8)	
**Antithrombotic therapy**		
Antiplatelet	7 (8.5)	0.699
Anticoagulant	7 (13)	
Combination	1 (12.5)	
**Suspension of antithrombotic therapy**	5 (15.6)	0.325
**Risk of intervention**		
Hemorrhagic risk		0.999
* Low*	15 (10.9)	
* Moderate*	0 (0)	
Thrombotic risk		0.442
* Low*	12 (10.1)	
* Moderate*	3 (18.8)	
* High*	0 (0)	

**Table 7:** Multiple logistic regression models for the analysis of event occurrence.

MODEL	Parameter	OR	95% CI		*p-Value*
			Lower Bound	Upper Bound	
**MODEL 1** *p*-value = 0.002 *Adjusted R2 = 0.133*	*Constant*	2.143			0.096
	***Inappropriate antithrombotic management***	6.650	2.109	20.968	0.001
**MODEL 2** *p*-value = 0.005 *Adjusted R2 = 0.251*	*Constant*	0.538			0.595
	***Inappropriate antithrombotic management***	4.661	1.330	16.332	0.016
	Age (≥75 years old)	0.528	0.151	1.846	0.317
	Gender (male)	0.231	0.057	0.934	
	Oral health procedure *				
	Non-surgical periodontal intervention	28.164	1.771	447.832	0.018
	Extraction	14.129	1.020	195.69.2	0.048
	Surgical intervention	13.283	0.988	178.602	0.051

* Reference category: Restorative intervention.

## Data Availability

Data generated during the current study are available from the corresponding author upon reasonable request.

## Supplementary Materials

The following supporting information can be downloaded at https://www.mdpi.com/article/10.3390/dj13050196/s1: Table S1. Patient distribution between the centers; Table S2. Reason (medical condition) for receiving antithrombotic therapy; Table S3. Frequencies and prevalence of each individual oral health procedure and the type of antithrombotic therapy; Table S4. Antithrombotic therapy management and suspension in relation to the type of oral health procedure; Table S5. Events associated with different oral health procedures.

## Abbreviations

The following abbreviations are used in this manuscript:

ASA	acetylsalicylic acid
BARC	Bleeding Academic Research Consortium
BMI	body mass index
CI	confidence interval
COPD	chronic obstructive pulmonary disease
ETEP	Etiology and Therapy of Periodontal and Peri-implant Diseases
INR	international normalized ratio
OHP	oral health procedure
OR	odds ratio
PMPR	professional mechanical plaque removal
SD	standard deviation
UCM	University Complutense of Madrid
