# Supplementary material for: Perioperative/Periprocedural Antithrombotic Management in Oral Health Procedures. A Prospective Observational Study
Source: Dent J (Basel). 2025 Apr 29;13(5):196. doi: 10.3390/dj13050196 (PMC12109777; doi:10.3390/dj13050196)
Supplement: Supplementary file 1 [file dentistry-13-00196-s001.zip › dentistry-3539015-supplementary.pdf]

Table S1. Patient distribution between the centers.

	n (%)
**Complutense University, Madrid**	64 (46.4)
**Rey Juan Carlos University, Madrid**	38 (27.5)
**Reina Sofía Hospital, Córdoba**	25 (18.1)
**Virgen Arrixaca Hospital, Murcia**	8 (5.8)
**Primary care health center, Córdoba**	1 (0.7)
**La Fe Hospital, Valencia**	1 (0.7)
**Primary care health center, Madrid**	1 (0.7)
**Total**	138 (100)

Table S2. Reason (medical condition) for receiving antithrombotic therapy.

	**n (%)**
**Atrial fibrillation**	36 (27.7)
**Mechanical heart valve**	4 (3.1)
**Venous thromboembolism**	2 (1.5)
**Recent thromboembolism**	1 (0.8)
**Recurrent thromboembolism**	1 (0.8)
**Ischemic heart disease**	47 (36.2)
**Cerebrovascular disease**	15 (11.5)
**Peripheral arterial disease**	2 (1.5)
**Primary prevention of CVD**	22 (16.9)

CVD: cardiovascular diseases

Table S3. Frequencies and prevalence of each individual oral health procedure and the type of antithrombotic therapy.

	**Restorative**	**Non-surgical periodontal interventions**		**Tooth extraction**	**Surgical interventions**							***p value***
		**Subgingival instrumentation**	**Supportive periodontal care visit**		**Periodontal surgery**	**Implant placement**	**Sinus lift**	**2nd phase implant surgery**	**Apical surgery**	**Pre-prosthetic surgery**	**Bone regeneration surgery**	
**Antiplatelet (n [%])**	3 (75)	31 (72.1)	7 (58.3)	17 (50)	9 (60)	13 (48.1)	2 (50)	0 (0)	0 (0)	0 (0)	0 (0)	*0.108*
Acetylsalicylic acid (ASA)	2 (50)	28 (65)	6 (50.1)	15 (44.1)	8 (53.3)	11 (40.8)	2 (50)	-	-	-	-	*0.979*
Clopidogrel	1 (25)	3 (7)	1 (8.3)	2 (5.9)	1 (6.7)	2 (7.4)	-	-	-	-	-	
**Anticoagulant (n [%])**	1 (25)	12 (27.9)	5 (41.7)	15 (44.1)	5 (3.3)	10 (37)	2 (50)	1 (100)	0 (0)	1 (100)	2 (100)	*0.108*
Acenocoumarin	-	6 (14)	3 (25)	5 (14.7)	2 (13.3)	3 (11.1)	2 (50)	1 (100)	-	1 (100)	1 (50)	*0.979*
Dabigatran	1(25)	1 (2.3)	0	5 (14.7)	2 (13.3)	2 (7.4)	-	-	-	-	-	
Rivaroxaban	-	-	1 (8.3)	1 (2.9)	-	-	-	-	-	-	-	
Apixaban	-	3 (7)	1 (8.3)	3 (8.9)	-	1 (3.7)	-	-	-	-	1 (50)	
Edoxaban	-	2 (4.7)	-	1 (2.9)	1 (6.7)	4 (14.8)	-	-	-	-	-	
**Combination (n [%])**	0 (0)	0 (0)	0 (0)	2 (5.9)	1 (6.7)	4 (14.9)	0 (0)	1 (100)	1 (100)	0 (0)	(0)	*0.108*
ASA and Edoxaban	-	-	-	-	-	1 (3.7)	-	-	-	-	-	*0.979*
ASA and Acenocoumarin	-	-	-	-	1 (6.7)	-	-	-	-	-	-	
ASA and Ticagrelor	-	-	-	2 (5.9)	-	1 (3.7)	-	-	-	-	-	
ASA and Clopidogrel	-	-	-	-	-	2 (7.4)	-	1 (100)	1 (100)	-	-	

Table S4. Antithrombotic therapy management and suspension in relation to the type of oral health procedure.

	Restorative	Non-surgical periodontal interventions		Tooth extraction	Surgical interventions							*p value*
		Subgingival instrumentation	Supportive periodontal care visit		Periodontal surgery	Implant placement	Sinus lift	2nd phase implant surgery	Apical surgery	Pre-prosthetic surgery	Bone regeneration surgery	
**Inappropriate antithrombotic management (n [%])**	2 (50)	3 (7)	1 (8.3)	8 (23.5)	2 (13.3)	4 (14.8)	0 (0)	1 (50)	0 (0)	0 (0)	1 (50)	*0.146*
**Suspension (n [%])**	2 (50)	4 (9.3)	0 (0)	13 (38.2)	2 (13.3)	8 (29.6)	1 (25)	1 (50)	0 (0)	0 (0)	1 (50)	*0.01*

Table S5. Events associated with different oral health procedures.

	Restorative	Non-surgical periodontal interventions		Tooth extraction	Surgical interventions							*p value*
		**Subgingival debridement**	**Supportive periodontal care visit**		**Periodontal surgery**	**Implant surgery**	**Sinus lift surgery**	**2nd phase implant surgery**	**Apical surgery**	**Pre-prosthetic Surgery**	**Bone regeneration surgery**	
**Yes (n [%])**	2 (50)	3 (7.0)	0 (0)	4 (11.8)	2 (13.3)	1 (3.7)	2 (50.0)	0 (0)	1 (100)	0 (0)	0 (0)	*0.048*
**Slight bleeding**	1 (25.0)	3 (7.0)	0 (0)	3(8.8)	2 (13.3)	1 (3.7)	2 (50.0)	0 (0)	1 (100)	0 (0)	0 (0)	*0.690*
**Bleeding requires suspension of antithrombotic treatment**	0 (0)	0 (0)	0 (0)	1 (2.9)	0 (0)	0 (0)	0 (0)	0 (0)	0 (0)	0 (0)	0 (0)	
**Myocardial infarction**	1 (25.0)	0 (0)	0 (0)	0 (0)	0 (0)	0 (0)	0 (0)	0 (0)	0 (0)	0 (0)	0 (0)	
